# The Importance of Patient-Specific Factors for Hepatic Drug Response and Toxicity

**DOI:** 10.3390/ijms17101714

**Published:** 2016-10-12

**Authors:** Volker M. Lauschke, Magnus Ingelman-Sundberg

**Affiliations:** Section of Pharmacogenetics, Department of Physiology and Pharmacology, Karolinska Institutet, SE-17177 Stockholm, Sweden; magnus.ingelman-sundberg@ki.se

**Keywords:** drug-induced liver injury, hepatotoxicity, liver disease, pharmacogenetics

## Abstract

Responses to drugs and pharmacological treatments differ considerably between individuals. Importantly, only 50%–75% of patients have been shown to react adequately to pharmacological interventions, whereas the others experience either a lack of efficacy or suffer from adverse events. The liver is of central importance in the metabolism of most drugs. Because of this exposed status, hepatotoxicity is amongst the most common adverse drug reactions and hepatic liabilities are the most prevalent reason for the termination of development programs of novel drug candidates. In recent years, more and more factors were unveiled that shape hepatic drug responses and thus underlie the observed inter-individual variability. In this review, we provide a comprehensive overview of different principle mechanisms of drug hepatotoxicity and illustrate how patient-specific factors, such as genetic, physiological and environmental factors, can shape drug responses. Furthermore, we highlight other parameters, such as concomitantly prescribed medications or liver diseases and how they modulate drug toxicity, pharmacokinetics and dynamics. Finally, we discuss recent progress in the field of in vitro toxicity models and evaluate their utility in reflecting patient-specific factors to study inter-individual differences in drug response and toxicity, as this understanding is necessary to pave the way for a patient-adjusted medicine.

## 1. Introduction

Interindividual differences in response to pharmacological treatment are a major health concern. Importantly, only 50%–75% of patients have been shown to react adequately to common pharmacological interventions [1], whereas the others exhibit either a lack of efficacy or suffer from adverse drug reactions (ADRs). Genetic, physiological (e.g., gender, age, concomitant diseases, starvation and circadian rhythm) and environmental factors (e.g., co-administered medications, diet, smoking behavior and environmental pollutants) can impact on drug response with genetic variability accounting for around 20%–30% of these interindividual differences [2]. Today, the most important biomarkers for drug treatment relate to genetic variants in the somatic genome of cancer cells, predicting the effect of oncological compounds. In contrast, the most prominent classes of genes affecting drug pharmacokinetics encode enzymes and transporters, modulating absorption, distribution, metabolism and excretion (ADME).

**Figure 1:**
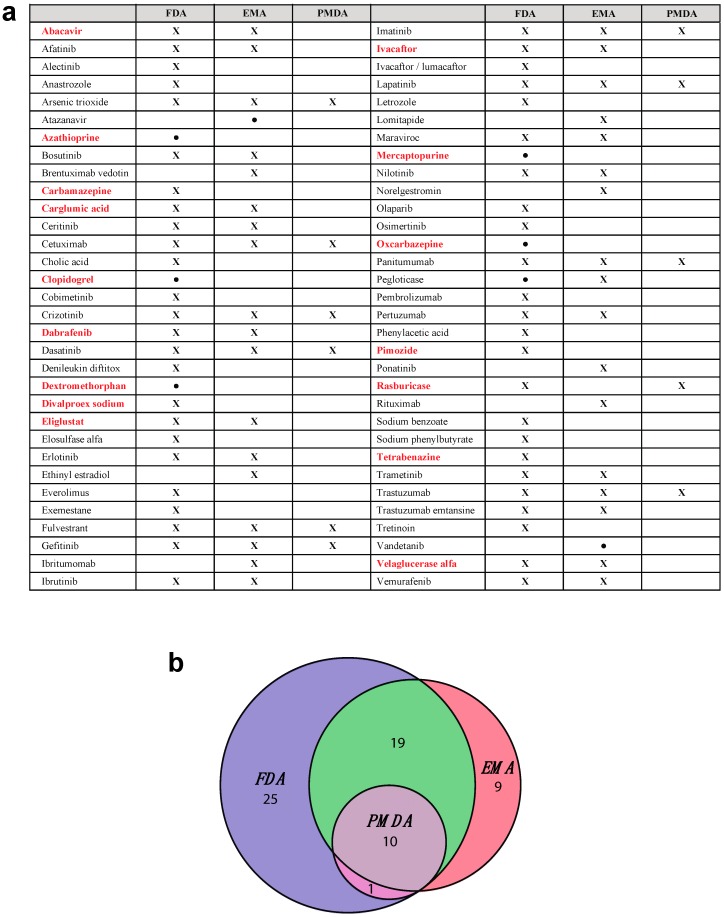
Drugs for which pharmacogenetic testing is recommended or required by major regulatory authorities: (**a**) Medications that require pharmacogenetic testing are indicated with “X”. If testing is only recommended, drugs are indicated with “●”. Requirements and recommendations by American (FDA), European (EMA) and Japanese (PMDA) regulatory authorities are shown. Note that only few medications (indicated in bold red) overlap with drugs for which prescribing action is recommended by the Clinical Pharmacogenetics Implementation Consortium (compare Table 1); (**b**) Venn diagram visualizing the overlap of drugs for which pharmacogenetic testing is required or recommended across FDA, EMA and PMDA.

The increasing understanding of genotype–drug response relationships led to a rise in numbers of drug labels with pharmacogenetic information issued by the US Food and Drug Administration (FDA) [3] and the European Medicines Agency (EMA) [4] targeted mainly at health care providers [5]. However, while thousands of biomarkers have been described in >150,000 scientific publications, currently only 24 genes are deemed pharmacogenetically actionable according to the Clinical Pharmacogenetics Implementation Consortium (CPIC; Table 1). Notably, this list only partly overlaps with the genetic testing requirements by American, European and Japanese regulatory agencies (Figure 1). Genotype-guided prescribing is only implemented for few drugs in the current clinical routine due to a variety of reasons, including: (i) problems in replicating identified associations, especially in the case of rare events; (ii) heterogeneous genetic nomenclature and non-standardized results reporting; as well as (iii) ethical; and (iv) regulatory considerations (reviewed in [6,7]). Therefore, overcoming these obstacles is of critical importance to further personalize pharmaceutical treatment, which could result in decreased morbidity and mortality for patients and a more efficient distribution of limited health-care resources.

Recent research indicated that the vast majority of genetic germline variants with importance for drug pharmacokinetics are rare with minor allele frequencies (MAF) below 1% [9,10,11]. These findings have important implications for the clinical application of pharmacogenomics, as they indicate that the phenotype of a patient regarding drug response cannot be reliably assessed by genotyping for few common variants and but rather that the entire genetic landscape in pharmacogenetic loci have to be analyzed comprehensively [12]. Besides genetic factors, inter-individual differences in drug response are caused by a multitude other parameters. In this review, we highlight the development of pharmacogenomic biomarkers and discuss concomitant liver diseases as factors that shape the response to administered medications. Lastly, we emphasize recent developments of cell models that are able to reflect these patient-specific factors and predict drug response and toxicity more accurately.

## 2. Socioeconomical Aspects of Drug Hepatotoxicity

Adverse reactions to medications account for approximately 6.5% of all hospital admissions and cause the death of 0.1%–0.2% of all hospitalized patients [13] with specific subpopulations being at even higher risk. In pediatric patients up to 39% of ADR-related hospitalizations have been found to be life threatening or fatal [14]. Similarly, studies from Europe and the US indicate that 10%–30% of geriatric hospital admissions are drug-related [15,16]. ADRs have been estimated to cost around 3,000 US$ per patient and amount to 5%–10% of annual hospital costs [17,18,19]. Combined costs for adverse medication-related events have been valued at 76.6 billion US$ in the United States alone [20], yet societal costs might be even higher due to underreporting of ADRs incidences [21] and the neglect of indirect costs [22].

Besides effects on patients and health care systems, ADRs are important cost drivers for the pharmaceutical industry, causing the termination of a plethora of drugs during clinical development stages due to safety liabilities with the liver being the second most common organ after the cardiovascular system to be involved in safety failures [23]. One formidable example is the toxicity seen with fialuridine (FIAU). FIAU, a nucleoside analog for therapy of hepatitis B infections did not show toxicity in preclinical test systems, yet, in clinical trials, 7 of 15 participants developed severe hepatic dysfunctions, five of whom died [24]. Another example is the termination of fasiglifam (TAK-875) in clinical phase 3 trials due to hepatic safety concerns [25]. Furthermore, in the last years, 2% of all FDA-approved new medications were endowed with boxed warnings due to hepatic ADRs [26] and three drugs were withdrawn in post-marketing stages for hepatotoxicity (bromfenac, troglitazone and pemoline).

## 3. Impact of Genetic Factors on Drug Metabolism

In recent decades, many genetic factors, such as single nucleotide polymorphisms (SNPs) or copy number variations (CNVs) have been identified that influence drug response and susceptibility to toxicity and entail a modification of drug dosing (Table 2). Major genetic determinants of hepatotoxicity due to altered drug metabolism include *DPYD* polymorphisms and 5-fluorouracil toxicity in treatment of solid carcinomas [27], variants in *TPMT* and hematological toxicity of 6-mercaptopurines for treatment of leukemia and morbus Crohn [28,29], gene duplications of *CYP2D6* and codeine toxicity [30] as well as the toxicity of the oncology compound irinotecan linked to indels in the *UGT1A1* promoter (UGT1A1*28) [31]. Furthermore, genetic variants have been reproducibly and mechanistically linked to drug efficacy, as exemplified by the effect of *CYP2C19* variants on voriconazole (CYP2C19*17) [32] and clopidogrel (CYP2C19*2) responsiveness [33].

One well-studied example of the impact of genetic polymorphisms on optimal dosing is illustrated by the impact of variants in *CYP2C9* and *VKORC1* on the metabolism of the anticoagulant warfarin that together account for approximately 30% of warfarin dose variability [34]. Furthermore, pharmacogenetic markers have been identified that affect drug efficacy, as evidenced by the relation of *CYP2C19* genotypes on the metabolism of proton-pump inhibitors, such as omeprazole and pantoprazole, which in turn affects gastric pH and the healing rate of peptic ulcers as well as of *Helicobacter pylori* infections [35,36]. Another interesting pharmacogenetic association has been identified for the manifestation of myopathies mostly upon high dose treatment with simvastatin (80 mg daily) in which the presence of a single SNP in the transporter *SLCO1B1* (rs4363657) can predict more than 60% of statin-induced myopathic ADRs [37]. For a more comprehensive overview of pharmacogenetic associations and their clinical translation, we refer to recent reviews that comprehensively summarized the progress in this field [38,39,40].

## 4. The Importance of Rare Variant Alleles for Pharmacogenetics

Strikingly, massive sequencing projects, such as the 1000 Genomes Project [41], the Exome Sequencing Project [42] and UK10K [43], revealed that the vast majority of genetic variants are rare with minor allele frequencies (MAFs) below 1%. These rare variants are mostly population-specific and not represented in genome-wide association studies (GWAS) or targeted genotyping platforms [44,45]. In genetic loci with importance for drug absorption, distribution, metabolism and excretion (ADME), recent studies indicated that more than 90% of all variants were rare and not currently assessed by pharmacogenetic genotyping [9,10,11]. These data indicate that comprehensive sequencing-based approaches are necessary to descry the true genetic makeup in pharmacogenes. Furthermore, the combined phenotypic impact of these rare variants on drug response was estimated to overall exceed 30% [11]. Interestingly, elegant twin-studies on the pharmacokinetics of metropolol and torsemide revealed that while approximately 90% of the metabolic capacity of these drugs is genetically determined, known variants in the responsible pharmacogenes *CYP2D6*, *CYP2C9*, and *SLCO1B1* only explained around 40% of the inter-individual differences [46]. These data corroborate the phenotypic importance of genetic variants beyond the well-characterized biomarkers, thus indicating that the assessment of rare genetic variability has to be incorporated into phenotypic predictions to be able to tailor treatment to the genotype of the individual patient within a precision medicine framework.

## 5. Mechanisms of Drug-Induced Hepatotoxicity

ADRs can be classified into reactions that are a direct consequence of the pharmacological action (e.g., hypotension with anti-hypertensive therapy and bleeding events with anti-coagulant treatment) of the drug and reactions in which toxicity and intended therapeutic mode of action differ (e.g., hepatic steatosis induced by the anti-epileptic drug valproic acid). The latter can be further subdivided into intrinsic ADRs with predictable rapid onset and typically dose-dependent severity (e.g., liver injury upon acetaminophen overdose) and idiosyncratic adverse reactions that occur with variable latency and where the risk to develop an ADR is not dependent on the dosing regimen but rather occurs only in few predisposed individuals (e.g., liver failure in patients treated with the anti-diabetic drug troglitazone). In the context of drug-induced liver injury (DILI), idiosyncratic reactions account for up to 10% of all DILI cases [47]. Chemically reactive metabolites (CRMs) are metabolic products that can result in mutagenicity or drug–drug interactions [48,49]. Furthermore, by covalently modifying proteins, CRMs of some compounds, including halothane [50,51] and diclofenac [52,53], can act as haptens and are recognized as a cause of idiosyncratic DILI reactions. Hence, efforts to reduce or eliminate such structural liabilities are routinely implemented in preclinical drug development pipelines. For an excellent critical overview of CRMs and the utility of structural alert analyses in preclinical development, we refer to the recent comprehensive review by Kalgutkar and Dalvie [54].

In the following section, we review key concepts in drug-induced hepatotoxicity. To this end, we focus on the role of mitochondria in cellular apoptosis and necrosis and highlight the role of the innate and adaptive immunity in DILI.

### 5.1. Mitochondrial Perturbations

**Figure 2:**
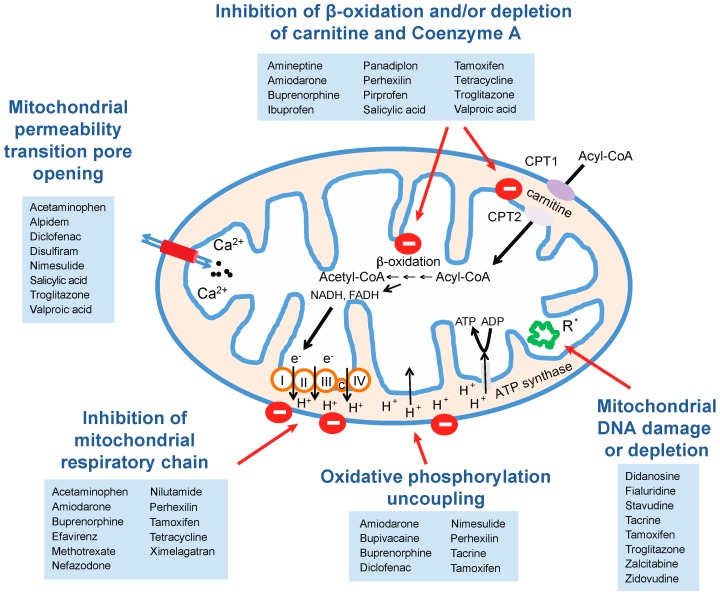
Schematic depiction of hepatotoxic drugs and their respective mitochondrial targets. Medications can exert toxic effects on mitochondria by targeting a variety of different processes, such as inhibition of mitochondrial respiratory chain components, uncoupling of oxidative phosphorylation or inhibition of β-oxidation and/or depletion of carnitine or coenzyme A. Some compounds, mostly antiretrovirals, can furthermore cause mitochondrial DNA depletion. Mitochondrial damage can result in opening of the mitochondrial permeability transition pore, causing loss of membrane potential, mitochondrial swelling and cell death by apoptosis or necrosis. The associated references are shown in Table 3.

Mitochondria are essential organelles that are involved in a variety of cellular processes. They generate the majority of cellular ATP in aerobic cells by oxidative phosphorylation, are the major site of fatty acid β-oxidation and oxidize pyruvate. Moreover, they are involved in apoptotic as well as necrotic cell death. Mitochondrial perturbations are a point of intersection of multiple different DILI mechanisms that can be as diverse as the direct toxicity seen with acetaminophen (APAP) [55] and immune-mediated liver injury due to tienilic acid [56] and are thus one of the major mechanisms underlying DILI [57]. Mitochondrial functionality can be impaired by directly inhibiting oxidative phosphorylation or fatty acid β-oxidation or by acting on mitochondrial DNA, transcripts or proteins (Figure 2). As a consequence of mitochondrial dysfunction, oxidative phosphorylation is uncoupled, ATP synthesis decreases and metabolic intermediates as well as pro-apoptotic molecules are released into the cytoplasm causing apoptosis or necrosis.

#### 5.1.1. Inhibition of Mitochondrial Respiration

**Figure 3:**
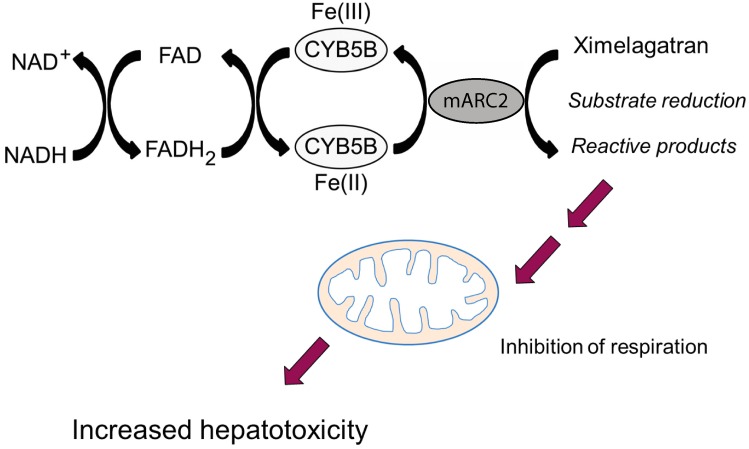
Proposed mechanisms of metabolic activation of ximelagatran. mARC2 in the outer mitochondrial membrane reduces ximelagatran to a reactive metabolite, which in turn inhibits mitochondrial respiration and causes hepatotoxicity.

The inhibition of mitochondrial respiration increases the formation of reactive oxygen species (ROS) by retaining electrons in upstream respiratory chain complexes. Furthermore, the oxidation of NADH to NAD+ is inhibited, which causes reduced capacity to oxidize pyruvate. As a result, pyruvate is primarily reduced to lactate and its buildup results in lactic acidosis. Furthermore, the paucity of NAD+ results in decreased β-oxidation and the accumulation of fatty acids causing steatosis [58]. Direct inhibition of the mitochondrial respiratory chain is caused e.g., by the non-nucleoside reverse-transcriptase inhibitor efavirenz, which is used for HIV treatment, and nefazodone, a triazolopyridine serotonin reuptake inhibitor. Efavirenz inhibits complex I of the respiratory chain in human hepatic cells in vitro, causing ATP depletion, compensatory upregulation of AMPK activity and increase in fatty acid uptake, leading to hepatic steatosis [59]. Nefazodone targets electron transport chain complexes I and IV, resulting in increased oxidative stress, glutathione depletion and hepatocellular necrosis [60]. Further examples of respiratory chain inhibition are the antiandrogen nilutamide, which inhibits NADH dehydrogenase (complex I) [61], and the antiarrythmic agent amiodarone, which causes inhibition of NADH dehydrogenase (complex I) and succinate dehydrogenase (complex II) in vitro [62]. In addition, we recently found the anticoagulant ximelagatran (Exanta) to decrease mitochondrial respiration after metabolic activation by mARC2 (also termed MOSC2; Figure 3) [63].

#### 5.1.2. Effects on Mitochondrial Lipid Metabolism

Drugs can also exert direct effects on β-oxidation by inhibition of the formation of long-chain fatty acids that can enter the mitochondria. Examples of β-oxidation-inhibiting drugs are troglitazone, amiodarone, valproic acid and salicylate. While all of these drugs inhibit uptake of fatty acids into mitochondria, their underlying mechanisms differ. Troglitazone inhibits long-chain acyl CoA synthetase [99], whereas amiodarone [92] and valproic acid [100] inhibit carnitine acyltransferase I (CPT1), the enzyme responsible for the transfer of the acyl group of long-chain fatty acyl-CoA molecules to carnitine, which constitutes an essential step in mitochondrial β-oxidation. In contrast, salicylic acid inhibits fatty acid elongation by depleting the cellular CoA-pool due to extensive metabolism of salicate to salicyl-CoA in vivo, thus resulting in impaired fatty acid elongation [97]. Downregulation of β-oxidation causes perturbations in the metabolic balance, as ketogenesis is impaired with the consequence that extrahepatic cells have to utilize glucose instead as energy source leading to hypoglycemic episodes during fasting periods [101].

#### 5.1.3. Mitochondrial DNA Damage and Inhibition of Mitochondrial Gene Expression

Some medications have been shown to act on mitochondrial DNA, transcripts or proteins. Dideoxynucleoside analogs, such as entecavir, used for treatment of chronic hepatitis B or zalcitabine and lamivudine for HIV therapy, constitute molecules that can be incorporated into a growing DNA strand, yet terminate DNA chain elongation due to the lack of a 3′-hydroxyl moiety [102,103]. Importantly, the incorporation of these analogs into replicating DNA strands depends on the specificity of responsible DNA polymerases. While the nucleotide analogs are not incorporated into nuclear DNA due to the specificity of nuclear DNA polymerases, they are incorporated into mitochondrial DNA (mtDNA) by the mitochondrial DNA polymerase γ [103]. As a result, these elongation terminating nucleotide analogs have to be removed by the proofreading activity of DNA polymerase γ, which markedly slows down replication of mtDNA, causes long-term mtDNA depletion and reduces expression of proteins in the mitochondrial respiratory complexes, which are all encoded within the mitochondrial genome [103]. Impaired biosynthesis of respiratory chain components causes the consequences outlined above, including increased ROS formation and reduced pyruvate oxidation [104].

Tetracycline inhibits mitochondrial protein translation, resulting in a stoichiometric imbalance of mitochondrial and nuclear gene products, thus disturbing proteostasis and resulting in unfolded protein response within mitochondria [105]. Clinically, this imbalance can manifest as microvesicular steatosis and liver failure [106] due to inhibition of β-oxidation [98] at high concentrations used in the past.

### 5.2. Immune-Mediated Toxicity

There is growing evidence that some drugs inducing DILI, constitute priming factors that initiate the recruitment and activation of immune cells to the liver and thereby cause hepatic injury (reviewed in reference [107]). The liver contains a variety of resident immune cells, including Kupffer and natural killer cells. During liver injury, the resident liver Kupffer cell populations are complemented by infiltrating macrophages expressing distinct surface markers [108]. Interestingly, liver resident Kupffer cells appear to have a liver protective effect, as evidenced by increased toxicity in Kupffer cell-depleted mice upon APAP exposure [109]. In contrast, inactivation of bone marrow-derived macrophages by gadolinium chloride protects from APAP toxicity (ALT levels 28 IU/L in treated mice vs. 6380 IU/L in untreated) [110].

Recent research elucidated various associations between HLA alleles and immune-mediated adverse drug reactions that can manifest in a variety of syndromes, such as drug hypersensitivity, systemic lupus erythematosis, Stevens–Johnson syndrome, toxic epidermal necrolysis, agranulocytosis or drug-induced liver injury (Table 4).

#### 5.2.1. Abacavir Hypersensitivity Syndrome (HSS)

Abacavir is a nucleoside-analog reverse-transcriptase inhibitor against HIV that is routinely used in combinations with other antiretroviral agents, such as lamivudine and zidovudine. Importantly, around 4% of patients show immune-mediated hypersensitivity to abacavir within the first six weeks of treatment, which mandates the discontinuation of abacavir therapy [138]. Importantly, abacavir hypersensitivity was reproducibly linked to the HLA-B*57:01 allele (odds ratio (*OR*) = 117) [111]. The abacavir parent molecule binds non-covalently to HLA-B*57:01 activating abacavir-specific T-cells, which then cause the systemic hypersensitivity syndrome [112] and introduction of a single variant (S116Y) into the HLA-B*57:01 allele by site-directed mutagenesis abrogated CD8^+^ T-cell recognition [113]. Double-blind, prospective, randomized clinical trials analyzing the utility of HLA-B*57:01 genotyping demonstrated a negative predictive value of 100% and a positive predictive value of 47.9% [139]. Furthermore, pharmacogenetic testing prior to treatment was mostly found to be cost-effective compared to subsequent treatment of hypersensitivity reactions but could depend on cost of genotyping and efficacy of alternative treatment [140,141,142]. Screening for the presence of HLA-B*57:01 has since become required by American (FDA) and European (EMA) regulatory authorities before starting abacavir therapy and thus presents a poster child for the successful implementation of pharmacogenetic research into clinical applications.

#### 5.2.2. Systemic Lupus Erythematosus (SLE)

An increased risk of SLE has been found in individuals exposed to hydralazine (adjusted *OR* = 6.6), minocycline (adjusted *OR* = 4.2) and carbamazepine (adjusted *OR* = 1.9) [143]. SLE is largely determined by heritable factors (>66%) with multiple risk alleles located in multiple genes of the major histocompatibility complex (MHC) region or the complement system [144] and associations of HLA-DRB1*03:01, *15:01, *08:01 and *14:01 with SLE have been consistently replicated [145,146,147,148]. Similarly, drug-induced lupus was linked to genetic predisposition, as evidenced by the correlation of minocycline-induced lupus with *HLA-DQB1* alleles with tyrosine at position 30 [115] and the association of *HLA-DR4* with SLE induced by hydralazine [114].

#### 5.2.3. Steven Johnson Syndrome (SJS) and Toxic Epidermal Necrolysis (TEN)

SJS and its more severe form TEN constitute adverse dermatological reactions to medications, which manifest in necrosis and detachment of the epidermis from lower skin layers. Drugs that cause SJS and TEN include carbamazepine, phenytoin, allopurinol and nevirapine. Hypersensitivity reactions to carbamazepine are strongly associated with the presence of HLA-B*15:02 in various Asian populations [116,117], whereas HLA-A*31:01 is a strong predictor in Northern Europeans and Japanese [118,119]. Similarly, HLA-B*15:02 was linked to SJS and TEN induced by phenytoin in Han Chinese and Thai [120,121]. Furthermore, SJS and TEN due to allopurinol treatment correlated with the HLA-B*58:01 allele across Asian and European populations [122,123,124,125]. Nevirapine is a non-nucleoside reverse transcriptase inhibitor that is part of a combinatorial HIV therapy in developing countries due to its low costs. However, around 20% of patients show adverse cutaneous reactions that range from localized rashes to SJS and TEN [149,150]. The HLA-B*35:05 allele significantly associated with skin rashes in Thais (*OR* = 9.3) but not in African (*OR* = 0.97), Caucasian (*OR* = 1.79) or northeast Asian (*OR* = 1.53) populations [126], whereas the HLA-C*04:01 allele was linked to nevirapine-induced SJS in Africans (*OR* = 17.5) [127].

#### 5.2.4. Clozapine-Induced Agranulocytosis

Clozapine, a dibenzodiazepine shows superior efficacy compared to other antipsychotics but its use is restricted due to high incidences (0.8% after 1 year) of clozapine-induced agranulocytosis [151]. Multiple genetic associations with alleles and single nucleotide polymorphisms (SNPs) in HLA alleles have been reported, including HLA-DRB1*04:02, DQB1*03:02, DQA1*0301, HLA-DR*02 and DQB1*05:02 as well as variants in *HLA-B* and *HLA-DQB1* [128,129]. However, even in the most comprehensive genetic study of clozapine-induced agranulocytosis performed to date, in which 163 cases were interrogated using genome-wide genotyping and whole-exome sequencing, odds ratios of the identified variants were low, rendering predictive clinical genotyping currently impracticable [129].

#### 5.2.5. Immune-Related Drug-Induced Liver Injury (DILI)

Several drugs have been described to cause immune-mediated liver damage. Liver injury due to flucloxacillin shows the strongest genetic HLA-DILI association identified to date with patients harboring the HLA-B*57:01 allele being at 80-fold higher [130], similar in magnitude to the hypersensitivity reactions observed upon abacavir treatment with the same allele (*OR* = 117) [111]. However, while only 13 patients would need to be tested to prevent one case of abacavir hypersensitivity, approximately 14,000 patients would need to be genotyped to prevent one flucloxacillin DILI case due to the low incidence of flucloxacillin DILI (8.5 in 100,000 flucloxacillin-treated patients) [152]. In addition, patients positive for HLA-B*57:01 (7% in Caucasians) would be denied flucloxacillin treatment although they would not develop DILI [153]. Thus, despite the strong genetic association, routine screening for HLA-B*57:01 should not be recommended for flucloxacillin therapy.

Co-amoxiclav is among the medications most commonly implicated in DILI, accounting for approximately 10% of DILI cases (after exclusion of acetaminophen cases) [154]. DILI due to amoxicillin-clavulanate significantly correlated with DRB1*15:01 in British populations with odds ratios between 2.3 and 9.3 [132,133,134]. Moreover, additional associations of co-amoxiclav hepatotoxicity with HLA-A*02:01 and HLA-B*18:01 were identified in a Spanish population [135]. Interestingly, HLA-A*30:02 and HLA-B*18:01 alleles were enriched in cases of hepatocellular injury, whereas HLA-DRB1*15:01 significantly associated with cholestatic and mixed DILI manifestations [155]. Corroborating the role of the immune system in amoxicillin-clavulanate, Kim et al. found that amoxicillin- and clavulanate-specific T-cells participate in amoxicillin-clavulanate-induced liver injury [156]. Similarly, risk of toxicity of the COX2-inhibitor lumiracoxib was significantly influenced by the common HLA haplotype HLA-DRB1*15:01-HLA-DQA1*01:02 (*OR* = 5.0) [136].

Susceptibility to DILI injury due to ticlopidine correlated significantly with the presence of the HLA-A*33:03 allele in Japanese patients (*OR* = 13) [137]. Ticlopidine is a prodrug that is metabolized mainly by CYP2B6 and CYP2C19 to its active metabolite [157]. Interestingly, studies in 114 individuals with ticlopidine-induced hepatotoxicity indicated that the HLA-A*33:03 related risk to develop DILI was further increased by gain-of-function variants in CYP2B6 (CYP2B6*1H and *1J; *OR* = 39), thus providing an interesting example of the intricate interplay of drug pharmacokinetics and the immune system in developing DILI [158].

Ximelagatran provides another example for a drug for which the immune system contributes to hepatotoxicity mechanisms has been proposed [131]. Eight percent of patients treated with ximelagatran showed dose-independent, delayed elevations of serum alanine aminotransferase (ALAT) levels resulting in the termination of the clinical development program of the drug [159]. Presence of the HLA-DRB1*07:01 allele was found to correlate with ximelagatran DILI (*OR* = 4.4) and its genetic distribution matches the geographic pattern of ALAT elevations (highest in Scandinavia and low in Asian populations) [131,159].

## 6. The Impact of Liver Diseases on Drug Response

Liver disease may have complex effects on drug clearance, biotransformation, and pharmacokinetics. Pathogenetic factors include alterations in intestinal absorption, plasma protein binding, hepatic extraction ratio, liver blood flow, porto-systemic shunting, biliary excretion, enterohepatic circulation, and renal clearance. For medications with intermediate to high hepatic extraction ratios, these effects can increase levels of bioavailable drug, mandating therapy at lower dosage. For instance, oral bioavailability of chlormethiazole and carvedilol is increased 12- and four-fold, respectively, in patients with liver cirrhosis [160,161]. Furthermore, shunting, sinusoidal capillarization and reduced liver perfusion can impair the functionality of oxidases, such as the CYP enzymes, due to reduced intracellular levels of molecular oxygen [162].

Activities of CYP2E1, CYP2D6, CYP1A2 and CYP2C19 were all found to decrease with increasing hepatic disease severity, their activities were differentially affected [163]. Activity of CYP2E1 was only lost in patients with decompensated cirrhosis, and also CYP2D6 function was relatively preserved. In contrast, CYP1A2 activity was found to decrease linearly with decreasing liver functions and metabolism of mephenytoin by CYP2C19 was already severely impaired by 63% in patients with mild liver disease (Pugh score 5 or 6) [163]. Similarly, activities of CYP3As were found to decrease in cirrhotic patients [164,165]. Corroborating these findings, hepatic expression of CYP1A2, CYP2E1 and CYP3A was found to be reduced in cirrhotic and severely cholestatic patients [166,167]. Consequently, these combined findings indicate that starting doses of CYP2D6, CYP2E1 and CYP3A4 substrates should be adjusted in patients with moderate or severe liver disease, whereas a dose reduction of CYP2C19 and CYP1A2 substrates should already be considered in milder forms of liver disease.

In contrast to the reduction of CYP activities, data on phase II metabolism in cirrhotic patients are conflicting. While some studies indicated that glucuronidation of benzodiazepines was not affected in cirrhotic patients [168,169], others showed reduced glucuronidation of morphine [170], zidovudine [171] and lamotrigine [172] in patients with advanced cirrhosis.

Besides cirrhosis, also other liver diseases can markedly impact on hepatic clearance and metabolism. Fisher et al. analyzed expression levels and metabolic capacities of CYPs during non-alcoholic fatty liver disease (NAFLD) progression [173]. Importantly, the authors found that activities of CYP1A2 and CYP2C19 decreased whereas metabolic capacities of CYP2A6 and CYP2C9 increased during progression from healthy livers to steatosis and non-alcoholic steatohepatitis (NASH). Similarly, CYP3A activity decreased in patients with hepatic steatosis [174]. While data on expression of CYP2E1 on the level of mRNA and protein are conflicting [173,175,176,177], enzymatic activities have been demonstrated to be increased in steatotic and NASH patients [175,178,179].

In addition to a reduction in CYP activity, multiple studies also described impaired phase II metabolism. Younossi et al. analyzed the liver proteomes of 98 obese patients and found, among others, a marked reduction of GSTM1, GSTM2 and GSTM4 (60% reduction) in patients with hepatic steatosis [180]. Furthermore, MGST2 was found to be downregulated in African NASH patients by 49% [181]. Interestingly, expression of efflux transporters of the ABC superfamily (*ABCC1*, *ABCC3-6*, *ABCB1*, *ABCG2*) increased with NAFLD progression from steatosis to NASH, whereas reduced glycosylation of MRP2 (encoded by *ABCC2*) resulted in reduced functional levels of this transporter at the apical plasma membrane [182]. Similarly, biliary transporters BSEP (*ABCB11*) and NTCP (*SLC10A1*) were found to be downregulated in NASH patients [183]. Altered transporter expression profiles can have direct impacts on drug disposition as demonstrated by altered metabolite concentrations in pediatric NASH patients upon a single APAP dose [184]. Specifically, APAP-glucuronide concentrations were increased in serum and urine, most likely due to reduced MRP2 and increased MRP3 activity, whereas APAP-sulfate levels were reduced, in agreement with previous reports [182,183].

Combined, the highlighted studies emphasize the pronounced impacts that hepatic diseases can have on drug ADME and shed light on the underlying molecular mechanisms on which these inter-individual differences are based on. This altered functionality of enzymes and transporters due to liver disease likely translates clinically into altered drug response.

## 7. Epigenetics and Inter-Individual Differences

Environmental as well as pathophysiological factors can moreover affect the epigenomic landscape. In seminal work by Murphy et al., the authors uncovered significant changes of DNA methylation patterns in liver biopsies that encompassed 69,247 DNA elements that correlated with progression of NAFLD [185]. Interestingly, epigenetic signatures matched expression changes in extracellular matrix remodeling factors, inflammatory molecules and ADME genes, including *CYP2C19* and *SLCO1B3*, fueling the hypothesis that altered DNA methylation in concert with histone modifications modulate gene activity and contribute to disease progression. Furthermore, epigenetic factors can provide mechanistic explanations for perturbations of drug metabolism in liver disease.

In the last decade, detailed epigenetic studies identified at least 60 ADME genes under epigenetic regulation and DNA methylation was in strong anti-correlation with gene expression [186]. The *CYP3A4* locus constitutes an impressive example for an epigenetic element involved in ADME gene expression. Activities of CYP3A4 can differ around 40-fold [187] and heritable factors have been estimated to account for 90% of this variability [188]. Interestingly, methylation of DNA elements in the proximal promoter or transcription factor binding sites correlated significantly with hepatic *CYP3A4* expression [189]. Recent research indicated that cytosine hydroxymethylation (5hmC) constitutes an additional epigenetic DNA modification, which is present on 0.5%–1% of total cytosine residues in adult human liver [190]. Interestingly, 5hmC levels have been found to correlate with the hepatic expression of ADME genes whereas no such correlation was detectable with conventional bisulfite sequencing, which is not capable of resolving between methylation and hydroxymethylation marks [191]. Combined, these data suggest a regulatory role of hydroxymethylation in liver development, homeostasis and metabolism.

However, while epigenetic and epigenomic studies convincingly indicate correlations between epigenetic alterations and gene expression changes, the question about causality remains. The advent of CRISPR/Cas9-based genomic editing tools that allow recruiting functional domains to loci of interest opens up possibilities to interrogate the impact of targeted epigenetic alterations on transcriptional outputs [192]. These developments fuel hopes that the epigenetic cause-consequence enigma can soon be tackled to provide understanding whether changes in gene expression profiles shape the epigenomic landscape, thereby reinforcing already established patterns or whether epigenetic factors are initial priming signals that render genetic loci permissive for transcription.

### In Vitro Toxicity Models That Reflect Patient-Specific Factors

In order to accurately predict hepatic drug response and toxicity, experimental model systems are needed that closely recapitulate and maintain the patient-specific factors outlined above. Primary human hepatocytes (PHH) are the most sensitive in vitro cell system and reflect molecular phenotypes of human hepatocytes in vivo most closely [193,194]. However, their physiological phenotypes are lost in conventional 2D monolayer cultures due to the lack of necessary biochemical cues and cell–cell interactions as well as non-physiological biophysical properties of the culture substratum, e.g., with regards to stiffness [195,196]. As a consequence, PHH lose expression of genes characteristic for mature hepatocytes within hours of culture and acquire fetal-like phenotypes [197,198,199]. To prevent this dedifferentiation a variety of advanced 3D hepatocyte culture methodologies have been developed (extensively reviewed in reference [200]). Hepatic cells can be cultured in stirred bioreactors, hanging drops or ultra-low attachment plates resulting in the formation of cellular aggregates termed spheroids. In spheroid culture, PHH remain viable and have been shown to retain high-level expression and metabolic capacity of hepatic genes [201,202,203]. Importantly, the inter-individual variability of hepatocytes isolated from different donors is maintained in spheroid cultures as evidenced by whole proteome analyses, which allows to emulate and study patient diversity in liver biology and drug response [203].

In addition to the maintenance of patient-specific molecular phenotypes in vitro, model systems are needed that incorporate hepatic diseases. To this end, the spheroid system can be expanded to mimic various hepatic pathologies. Drug-induced cholestasis can be replicated as exemplified by treatment with chlorpromazine resulting in significant downregulation of *ABCB11*, encoding the bile acid transporter BSEP, and a marked accumulation of intra-cellular bile acids [203]. Moreover, 3D systems present pathophysiologically relevant model systems to study the hepatic manifestations of metabolic syndrome and type 2 diabetes mellitus (T2DM). Hepatocytes in such models can remain sensitive to insulin signaling for multiple weeks in normoglycemic conditions, whereas hepatocellular steatosis is induced under elevated glucose exposure [204]. Furthermore, as hepatocytes can be co-cultured with various non-parenchymal cells (NPCs), including Kupffer, stellate and biliary cells, advanced 3D models offer the potential to be useful in simulating NAFLD progression from steatosis to NASH and fibrosis [203].

Combined, advancements in hepatocyte culture technologies allow capturing liver biology, hepatic metabolism and liver pathology more and more accurately, thus opening possibilities to improve the quality of preclinical toxicity assessments in drug development. Furthermore, given the appropriate culture conditions, the spheroid systems indicated above constitutes a suitable tool to study the factors underlying the inter-individual variability in drug response. As such, they might become viable options to perform small “clinical trials” in vitro before entering clinical development stages with high cost-saving potentials for the pharmaceutical industry and reduced risks for trial participants.

## 8. Conclusions

Personalized medicine, defined as the individualization of prevention, diagnosis and treatment, is conceptually nothing new. However, it has received growing attention due to the extended opportunities that came with the recent progress in sequencing technology and data interpretation, expanding the patient-specific factors that can be considered from classical parameters such as weight, age and clinical chemistry readouts to complex genetic predictors.

The liver is an organ of central importance in the individualization of treatment due to its critical role in drug metabolism and a plethora of associations of genotypes with drug metabolism and/or toxicity have by now been convincingly described. Most commonly, these variants can be found in ADME genes modulating expression levels or resulting in increased or decreased activity of their respective gene products, thereby changing absorption, bioactivation, detoxification or excretion of the administered medication, resulting in reduced efficacy or increased toxicity. Perturbation of mitochondrial functions is a common mechanism of drug-induced toxicity. It can occur due to inhibition of mitochondrial respiration, inhibition of lipid metabolism or damage to mitochondrial DNA (Figure 2). Furthermore, drugs can directly or indirectly open the mitochondrial permeability pore, thus inducing apoptosis.

Besides impacting drug metabolism, genetic variants can also modulate the risk of immune-mediated toxicity reactions. This relationship of immune system and drug toxicity is best understood for the hypersensitivity reactions upon abacavir treatment that occur exclusively in patients harboring HLA-B*57:01, *HLA-DR7*, and *HLA-DQ3* (positive predictive value of 100% and a negative predictive value of 97%) [111], in which abacavir has been shown to non-covalently interact with HLA-B*57:01, triggering a CD8^+^ T-cell response [112]. However, a growing body of literature indicates that pharmacogenetic associations with variants in major histocompatibility complex (MHC) genes are more common (Table 2).

Liver diseases are another important factor that can influence drug metabolism and clearance and, accordingly, treatment response. Interestingly, drug-metabolizing enzymes were differentially sensitive towards liver diseases, as evidenced by drastically reduced CYP2C19 activity in patients with mild liver disease, whereas CYP2E1 activity only decreased in decompensated cirrhosis [163]. Pathologies, dietary and environmental factors cause alterations of the epigenomic landscape, which has spurred the exploration into epigenetic biomarkers that could predict drug response or treatment outcome ideally from bodily fluids. Some epigenetic biomarkers, such as hypermethylated fragments of *SEPT9* in plasma for colorectal cancer diagnosis (sensitivity 90%, specificity 88%, reference [205]) and *APC*, *GSTP1* and *RARB2* promoter hypermethylation in urine for prostate cancer detection (sensitivity 69%, specificity 82%, reference [206]) have shown promise for disease diagnosis. They have been made commercially available (e.g., ProCaM™ and ^m^SEPT9) but, so far, have not been adopted in routine clinical screening programs. In contrast, to our knowledge no blood-based biomarker predictive of drug response has been identified, thus suggesting that non-invasive pharmacoepigenomics will not be clinically implemented in the near future.

Currently, only 1.3% of candidate drugs (CDs) entering clinical trials acquire regulatory approval, many due to safety concerns [23,207]. Importantly, increasing confidence in preclinical safety profiles of a CD drastically decreases the likelihood of termination of the respective project in clinical stages due to safety concerns [23]. Combined, these data suggest that current preclinical systems, such as conventional 2D cell culture systems and laboratory animals, do not accurately mimic human drug response. Hence, more predictive preclinical systems are required to increase success rates in clinical stages of the drug development pipeline, resulting in decreased morbidity and mortality of trial participants and decreased costs for the trial sponsor. To address this need, a plethora of advanced 3D cell culture systems were developed, some of which represent significant advancements by enabling chronic toxicity assessments at exposure levels that approximate therapeutic concentrations. While hepatocytes isolated from different patients can retain their inter-individual differences in 3D systems and have been successfully applied to mimic hepatocellular injuries due to mitochondrial toxicity and metabolic alterations in diseased conditions, the capture of idiosyncratic immune-mediated responses remains currently unpredictable.

While results obtained with these systems are encouraging, the field requires the standardization of protocols and systematic validation studies, ideally performed in a joint cross-pharma setting, to facilitate wider adoption in academia and industry with the long-term aim of acceptance by regulatory bodies.

## Figures and Tables

**Table 1:** Overview of actionable pharmacogenetic gene–drug pairs. Data obtained from reference [8]. In total, 86 actionable gene drug pairs (24 unique genes and 73 unique drugs) are listed for which a change in prescribing is recommended based on genetic makeup of the patient (Actionable label and CPIC levels A or B).

Gene	Actionable Pairs	Medications
*CYP2D6*	20	Amitriptyline, codeine, fluvoxamine, nortriptyline, tramadol, aripiprazole, atomoxetine, clomipramine, desipramine, doxepin, imipramine, protriptyline, trimipramine, vortioxetine, iloperidone, perphenazine, dextromethorphan, eliglustat, pimozide, tetrabenazine
*DPYD*	2	Capecitabine, fluorouracil
*HLA-A*	1	Carbamazepine
*CACNA1S*	4	Desflurane, isoflurane, sevoflurane, succinylcholine
*RYR1*	4	Desflurane, isoflurane, sevoflurane, succinylcholine
*UGT1A1*	2	Irinotecan, belinostat
*HLA-B*	4	Phenytoin, oxcarbazepine, abacavir, carbamazepine
*TPMT*	3	Thioguanine, azathioprine, mercaptopurine
*CYP2C19*	8	Voriconazole, citalopram, dexlansoprazole, doxepin, esomeprazole, pantoprazole, carisoprodol, clopidogrel
*CYP2C9*	2	Warfarin, celecoxib
*VKORC1*	1	Warfarin
*IFNL3*	1	Peginterferon α-2b
*G6PD*	22	Chloroquine, chlorpropamide, dapsone, glibenclamide, glimepiride, glipizide, mafenide, methylene blue, nalidixic acid, nitrofurantoin, norfloxacin, primaquine, probenecid, quinine, sodium nitrite, sulfadiazine, sulfasalazine, erythromycin, sulfisoxazole, dabrafenib, pegloticase, rasburicase
*HPRT1*	1	Mycophenolic acid
*ABL2*	1	Valproic acid
*ASL*	1	Valproic acid
*ASS1*	1	Valproic acid
*CPS1*	1	Valproic acid
*NAGS*	1	Valproic acid
*OTC*	1	Valproic acid
*POLG*	2	Valproic acid, divalproex sodium
*CFTR*	1	Ivacaftor
*NAGS*	1	Carglumic acid
*GBA*	1	Velaglucerase α

**Table 2:** Pharmacogenetic associations and their impact on dosing and prescribing. Dosing recommendations were gathered from the Clinical Pharmacogenetics Implementation Consortium (CPIC), the Royal Dutch Association for the Advancement of Pharmacy—Pharmacogenetics Working Group (DPWG) and the French National Pharmacogenetics Network together with the Group of Clinical Onco-pharmacology. DPD = dihydropyrimidine dehydrogenase; TPMT = thiopurine *S*-methyltransferase.

Drug	Gene	Activity Level (Exemplary Genotypes)	Pharmacological Consequence	Dosing Recommendation
Fluoropyrimidines	*DPYD*	Intermediate DPD activity (*1/*2A, *1/*13)	Decreased fluoropyrimidine catabolism and increased levels toxic metabolites	At least 50% initial dose reduction
		DPD deficiency (*2A/*2A, *13/*13)		Select alternate drug
Mercaptopurine	*TPMT*	Intermediate TPMT activity (*1/*2, *1/*3A, *1/*3B, *1/*3C, *1/*4)	Increased levels of cytotoxic TGN metabolite	Reduction to 30%–70% of normal starting dose
		TPMT deficiency (*3A/*3A, *2/*3A, *3C/*3A, *3C/*4, *3C/*2, *3A/*4)		Drastic dose reduction to <10% or consider alternative therapy
Codeine	*CYP2D6*	Ultrarapid metabolizer (*1/*1xN, *1/*2xN)	Increased formation of morphine	Select alternate drug
		Intermediate metabolizer (*5/*41, *4/*10)	Reduced formation of morphine	Dosage according to label. If no response, select alternate drug
		Poor metabolizer (*4/*4, *4/*5, *5/*5, *4/*6)	Drastically reduced formation of morphine	Select alternate drug due to lack of efficacy
Irinotecan	*UGT1A1*	Intermediate UGT1A1 activity (*1/*28, *1/*37)	Reduced glucuronidation of active metabolite SN-38	Standard dose with rigorous clinical surveillance
		Strongly reduced UGT1A1 activity (*28/*28, *37/*37)		Dose reduction of 30% for standard dose, no dose intensification
Clopidogrel	*CYP2C19*	Ultrarapid metabolizer (*1/*17, *17/*17)	Increased formation of active metabolite, decreased platelet aggregation	Standard dose
		Intermediate metabolizer (*1/*2, *1/*3, *2/*17)	Reduced formation of active metabolite, increased platelet aggregation	Select alternate drug
		Poor metabolizer (*2/*2, *3/*3, *4/*4, *5/*5, *6/*6, *7/*7, *8/*8)		Select alternate drug
Omeprazole	*CYP2C19*	Ultrarapid metabolizer (*1/*17, *17/*17)	Increased metabolic inactivation to 5-hydroxyomeprazole	Increase dose 2–3-fold for *H. pylori* eradication therapy
		Intermediate metabolizer (*1/*2, *1/*3, *2/*17)	Decreased metabolic inactivation to 5-hydroxyomeprazole	Standard dose
		Poor metabolizer (*2/*2, *3/*3, *4/*4, *5/*5, *6/*6, *7/*7, *8/*8)		Standard dose
Simvastatin	*SLCO1B1*	Intermediate SLCO1B1 activity (*1a/*5, *1a/*15, *1a/*17, 1b/*5, *1b/*15, *1b/*17)	Decreased hepatic simvastatin uptake	High simvastatin doses (80 mg/day) not recommended, consider alternative statin
		Strongly reduced SLCO1B1 activity (*5/*5, *15/*15, *17/*17)		

**Table 3:** References describing the mitochondrial effect of the drugs highlighted in Figure 2.

Pathway	Drug	Reference
Mitochondrial permeability transition pore opening	Acetaminophen	Kon et al., 2004 [64]
	Alpidem	Berson et al., 2001 [65]
	Diclofenac	Masubuchi et al., 2002 [66]
	Disulfiram	Balakirev et al., 2001 [67]
	Nimesulide	Mingatto et al., 2000 [68]
	Salicylic acid	Trost et al., 1996 [69]
	Troglitazone	Tirmenstein et al., 2002 and Lim et al., 2008 [70,71]
	Valproic acid	Trost et al., [69]
Inhibition of mitochondrial respiratory chain	Acetaminophen	Meyers et al., 1988, Donnelly et al., 1994 and Lee et al., 2015 [72,73,74]
	Amiodarone	Fromenty et al., 1990 [62]
	Buprenorphine	Berson et al., 2001 [75]
	Efavirenz	Blas-Garcia et al., 2010 [59]
	Methotrexate	Yamamoto et al., 1988 [76]
	Nefazodone	Dykens et al., 2008 [60]
	Nilutamide	Berson et al., 1994 [61]
	Perhexillin	Deschamps et al., 1994 [77]
	Tamoxifen	Cardoso et al., 2001 and Larosche et al., 2007 [78,79]
	Tetracycline	Pious and Hawley, 1972 [80]
	Ximelagatran	Neve et al., 2015 [63]
Oxidative phosphorylation uncoupling	Amiodarone	Fromenty et al., 1990 [62]
	Bupivacaine	Dabadie et al., 1997 [81]
	Buprenorphine	Berson et al., 2001 [75]
	Diclofenac	Ponsoda et al., 1995 and Syed et al., 2016 [82,83]
	Nimesulide	Mingatto et al., 2002 [84]
	Perhexillin	Deschamps et al., 1994 [77]
	Tacrine	Berson et al., 1996 [85]
	Tamoxifen	Cardoso et al., 2001 [78]
Mitochondrial DNA depletion	Didanosine	Walker et al., 2004 [86]
	Fialuridine	McKenzie et al., 1995 [87]
	Stavudine	Walker et al., 2004 [86]
	Tacrine	Mansouri et al., 2003 [88]
	Tamoxifen	Larosche et al., 2007 [79]
	Troglitazone	Rachek et al., 2009 [89]
	Zalcitabine	Walker et al., 2004 [86]
	Zidovudine	De la Asuncion et al., 1999 [90]
Inhibition of β-oxidation and/or depletion of carnitine and Coenzyme A	Amineptine	Le Dinh et al., 1988 [91]
	Amiodarone	Kennedy et al., 1996 [92]
	Buprenorphine	Berson et al., 2001 [75]
	Ibuprofen	Fréneaux et al., 1990 and Baldwin et al., 1998 [93,94]
	Panadiplon	Ulrich et al., 1998 [95]
	Perhexillin	Deschamps et al., 1994 and Kennedy et al., 1994 [77,92]
	Pirprofen	Genève et al., 1987 [96]
	Salicylic acid	Deschamps et al., 1991 [97]
	Tamoxifen	Larosche et al., 2007 [79]
	Tetracyclin	Fréneaux et al., 1988 [98]
	Troglitazone	Fulgencio et al., 1996 [99]
	Valproic acid	Aires et al., 2010 [100]

**Table 4:** Pharmacogenetics of immune-mediated adverse drug reactions. NSAID = non-steroidal anti-inflammatory drug; HSS = Hypersensitivity syndrome; SJS = Stevens–Johnson syndrome; TEN = toxic epidermal necrolysis; DILI = Drug-induced liver injury.

Drug	Class of Drug	HLA Allele	Adverse Reaction	Reference
Abacavir	Antiretroviral	B*57:01, DR7 and DQ3	HSS	[111,112,113]
Hydralazine	Vasodilator	DR4	SLE	[114]
Minocycline	Antibiotic	DQB1 alleles with tyrosine at position 30	SLE	[115]
Carbamazepine	Anticonvulsant	B*15:02 and A*31:01	HSS and SJS/TEN	[116,117,118,119,120]
Phenytoin	Anticonvulsant	B*15:02	SJS/TEN	[120,121]
Allopurinol	Uricosuric	B*58:01	SJS/TEN	[122,123,124,125]
Nevirapine	Antiretroviral	B*35:05 and C*04:01	SJS/TEN	[126,127]
Clozapine	Antipsychotic	Multiple	Agranulocytosis	[128,129]
Flucloxacillin	Antibiotic	B*57:01	DILI	[130]
Ximelagatran	Anticoagulant	DRB1*07:01 and DQA1*02:01	DILI	[131]
Co-amoxiclav	Antibiotic	DRB1*15:01 and A*02:01 and B*18:01	DILI	[132,133,134,135]
Lumiracoxib	NSAID	DRB*15:01 and DQA*01:02	DILI	[136]
Ticlopidine	Anticoagulant	A*33:03	DILI	[137]
